# Pollution and contamination assessment of heavy metals in the sediments of Jazmurian playa in southeast Iran

**DOI:** 10.1038/s41598-020-61838-x

**Published:** 2020-03-16

**Authors:** Mahboube Shirani, Keramat Nezhad Afzali, Sayka Jahan, Vladimir Strezov, Mojtaba Soleimani-Sardo

**Affiliations:** 1 Department of Chemistry, Faculty of Science, University of Jiroft, Jiroft, P. O. Box 7867161167 Iran; 2 Department of Geography, Faculty of Humanities and Literature, University of Jiroft, Jiroft, P. O. Box 7867161167 Iran; 3 Department of Earth and Environmental Sciences, Faculty of Science and Engineering, Macquarie University NSW 2109, Sydney, Australia; 4 ARC Research Hub for Computational Particle Technology, Macquarie University, Sydney, New South Wales 2109 Australia; 5 Department of Environmental Science and Engineering, Faculty of Natural Resources, University of Jiroft, Jiroft, P. O. Box 7867161167 Iran

**Keywords:** Element cycles, Environmental impact, Environmental monitoring

## Abstract

Jazmurian playa was an ephemeral lake with a large catchment in southeast Iran, which dried up over the last 10 years as a result of prolonged drought. As the lake was recipient of incoming industrial water with trace metals deposited to the sediment, the dust is the cause of environmental concern of the region and requires evaluation and better management. The aim of this study was to evaluate the environmental and ecological pollution of Jazmurian playa. Hence, 24 collected surface sedimentary samples were analyzed with ICP-OES. The environmental pollution indices including degree of contamination (Cd), geoaccumulation index (Igeo), enrichment factor (EF), pollution load index (PLI) and potential ecological risk (PER) were determined. The study revealed moderately to strongly pollution levels of Pb, Ni and Al, while Dy, Pb, Y, Yb, Sm, Te, U, Cu, Mn, Sc represented moderate pollution. The EF values indicated four sites were highly enriched with Dy, Pb and Ni. The PER results showed high risk for four sites and considerable risk for others. Cluster analysis illustrated interconnection between the contaminants and the sites with major pollution at six sites. Obviously, climate change has considerable complex environmental impacts through transformation of local water and sediment pollution problem.

## Introduction

One of the currently most challenging environmental concerns is heavy metals pollution of soil and water sources, which can result in environmental toxicity and adverse impacts on human health and ecosystems^1–3^. The contamination in soil and water can transfer to the food chain and bioaccummulate in body. Human exposure to heavy metals occurs through consumption of contaminated water or food^4,5^, or through respiration by inhaling atmospheric particles enriched with heavy metals. Accumulation of the heavy metals in body even at trace levels causes neurological disorders, hormone imbalance, cardiovascular failure, kidney diseases, infertility, hair loss, endocrine disorders, respiratory and digestive problems, and cancer^6–8^. A range of industrial activities discharge their wastewaters into the rivers, which then deposit and enrich in the river sediments. River sediments are typically used to assess pollution of heavy metals using a range of defined pollution indices^9–11^.

In some countries such as Iran, located in western Asia and comprising a land area of 1.65 million km^2^ with about 20% covered by deserts^12^, due to the decrease in rainfall and climate warming most rivers have dried up in recent years, exposing the metal enriched sediments to atmosphere, resulting in air pollution through episodes of dust storms^13,14^. Jazmurian playa is located in the Sistan basin, which has changed to a desert over time, and serves as an example of climate change driven transformation of water pollution problem to an atmospheric pollution hazard. The sediments in playas have been a subject of natural and anthropogenic sources of waterborne metal deposition. The Jazmurian area is surrounded by many mines, such as arsenic, gold, chromium, copper, iron, manganese, antimony and titanium. Hence, sulphuric acid resulting from acid mine drainage contributed to emission of metal ions through the region, causing water and sediment contamination and accumulation over time^15^, which concentrated and dried on the surface of the playas^16,17^. Sedimentation reduces the playa volume and finally leads to completely drying out of the playas. This process causes accumulation of heavy metals on the sediments of the playa^18,19^. Wind easily transfers the polluted sediment particles as dust storms and the pollution emission occurs naturally. Hence, analysis of the surface sediments on the playa can reasonably reflect the composition, toxicity and the pollution rate of dust storms^20,21^. Although, some studies have been reported in this respect, to the best of our knowledge, there are currently no published papers discussing the heavy metal analysis of the sediment of Jazmurian playa, This is the first study to examine the contamination of sediments of a basin dried due to climatic variations and human activities.

In this paper, the environmental and ecological pollution is studied over Jazmurian playa using sediment sampling and analysis of heavy metals. For this purpose, the pollution indices including degree of contamination (C_d_), degree of contamination index (mC_d_), geoaccumulation index (Igeo), enrichment factor (EF), pollution load index (PLI), and potential ecological risk index (PER) are investigated over Jazmurian playa.

## Materials and methods

### Description of study area

**Figure 1:**
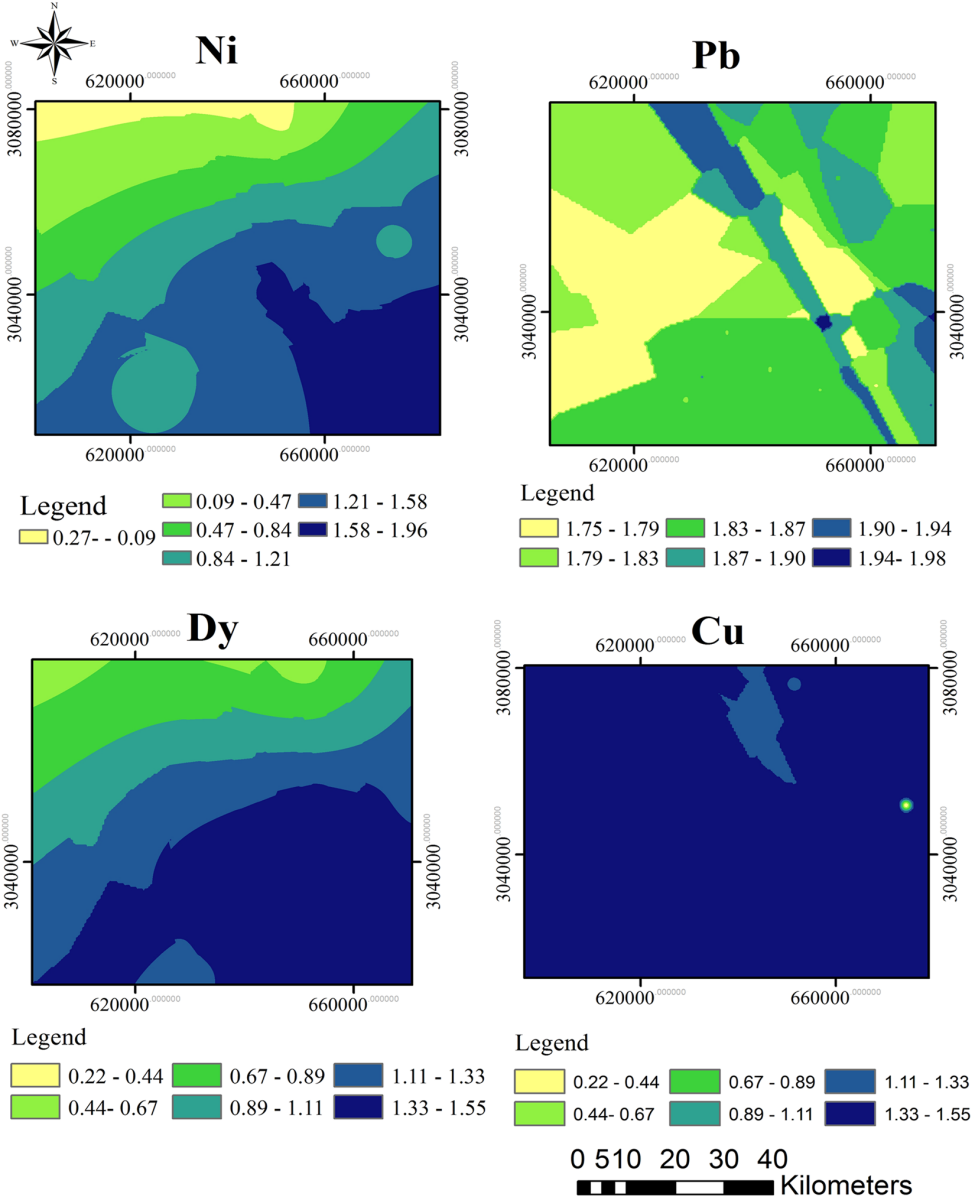
The geographic location of study area created by ArcGIS 10.5, https://www.arcgis.com/index.html.

Jazmurian basin, as an endorheic basin, is a tectonic depression in southeast of Iran with an area of approximately 69600 km^2^ located at 26°35′ to 29°28′N and 56°18′ to 61° 25′ E (Fig. 1). Hamun Jazmurian lake is a seasonal lake located at the center of the basin between the provinces of Sistan Va Baluchestan and Kerman, surrounded by mountain ranges Jebal-e Barez on the north and Makran on the south. The Jazmurian lake is covered by aggregations of sediments accumulated and deposited with a uniform texture. The studied area, which is about 1600 km^2^ and was selected as representative of the desert area of Jazmurian Playa, has the highest potential for dust storm due to the sediment exposure to atmospheric erosion and suspension. The depression is surrounded by rough rocky and mountainous area with about 1000–3000 meters altitude, from where rivers and surface water originate and then move into the depression^12^. Volcanic mountains in the northeast (Jebal Barez) separate Jazmurian from the Loot Desert. The highlands in the west and southeast of the basin are made up of intrusive and extrusive igneous rocks which have covered the upper Cretaceous rocks. Along the east, there are evaporative Miocene faulted rocks which belong to the upper red formations. Along its southern part the Makranophiolite mélange separates Jazmurian from Oman Sea. The average annual rainfall in Jazmurian is more than 200 mm in the northwest altitudes, 150 mm in the eastern part immediately after Iranshahr, and less than 100 mm in its southwest part. In the central part of Jazmurian at the height of 360 meters there is a seasonal playa with a length of 65 km and width of 45 km which sinks into water in winter and then dries out in summer. However, due to the prolonged droughts during the last 10 years, the playa has been devoid of any water. The area of the playa is 3775 square kilometers which is five percent of the whole catchment. The study area and the satellite image are illustrated in Fig. 1 as obtained by ArcGIS version 10.5.

### Sample collection procedure

In the present study 24 sediment samples were collected from Jazmurian playa on February 2018 and 23 trace elements, including Fe, Co, Cu, Al, Dy, Pb, Sm, U, Ni, Cd, Cr, Se, Ba, Ti, Mn, V, Sc, Zn, As, Rb, Te, Y and Yb were analysed by ICP-OES. In order to evaluate the sediment quality of the Jazmurian playa and consider the contamination assessment of the area, different pollution factors including degree of contamination (C_d_), degree of contamination index (mC_d_), geoaccumulation index (Igeo), enrichment factor (EF), pollution load index (PLI) and potential ecological risk index (PER) were calculated and obtained.

**Figure 2:**
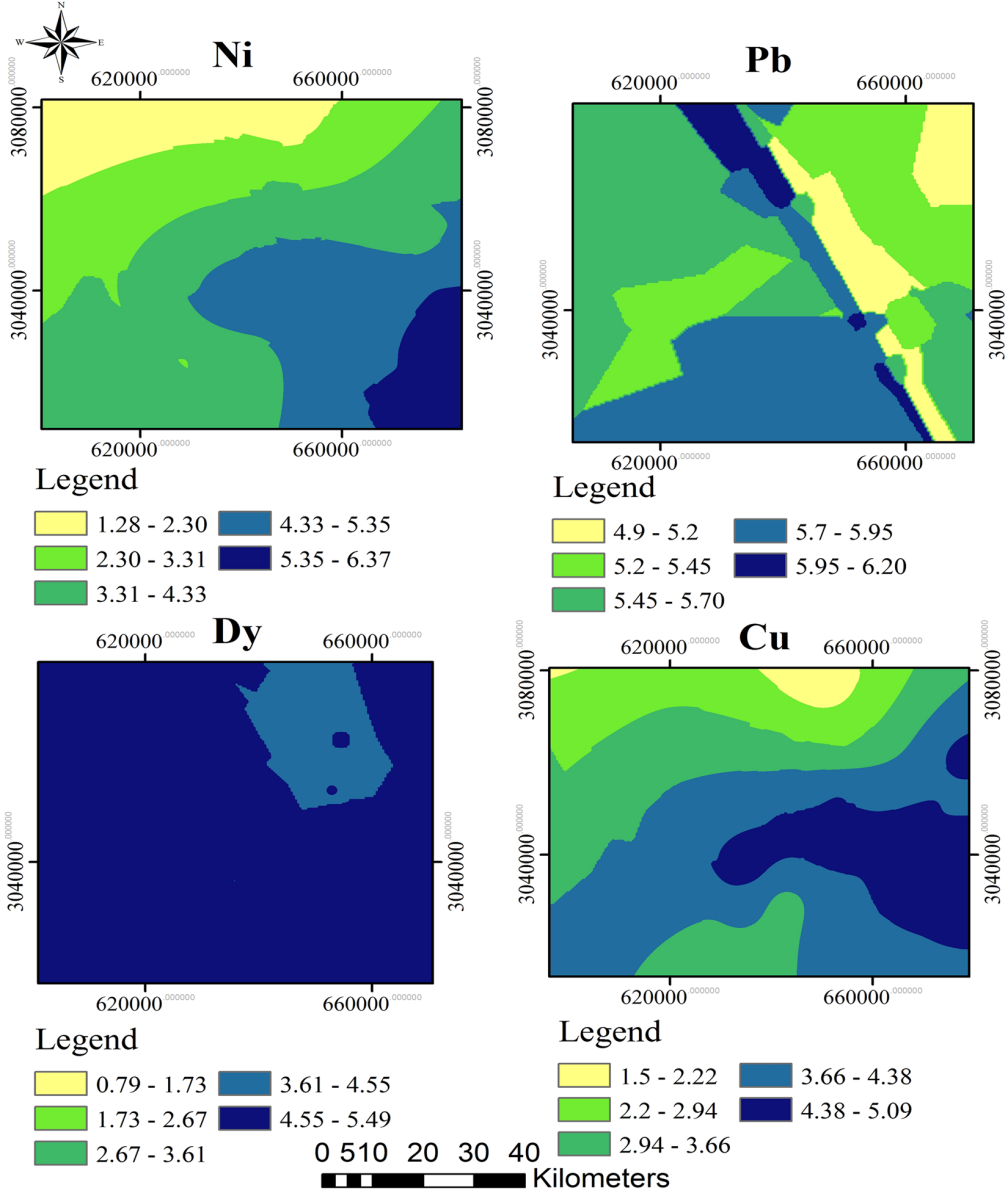
Surface sediments sampling area of Jazmurian playa.

**Figure 3:**
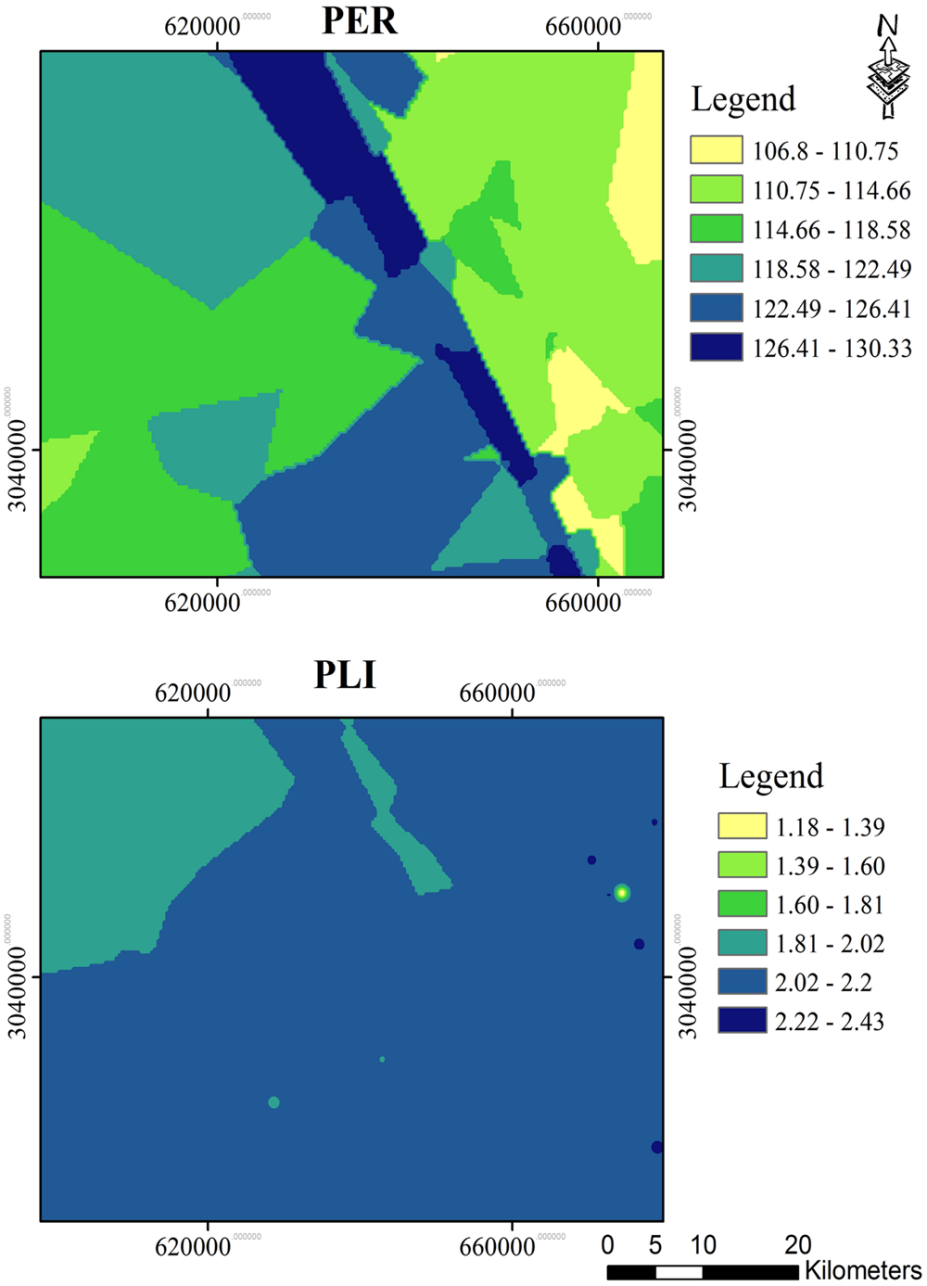
Geological map of the study area (Qft1 = High level piedmont fan and valley terrace deposits; Qft2= Low level piedment fan and valley terrace deposits; PlQc = Fluvial conglomerate, Piedmont conglomerate and sandstone; Qs = Sand dunes and sand sheet; Qm = Swamp and marsh; PlQabv = Andesite, andesitic basalt and olivine basalt (Bazman Volcanism); Qs = Sand dunes and sand sheet; om2 = Tectonized association of pelagic limestone, radiolarian chert, radiolarian shale with basic volcanic and intrusive rocks of ophiolitic rocks; EOf = Rytmically bedded sandstone and shale with volcanoclastic sandstone, minor limestone and tuff; Plms = Marl, shale, sandstone and conglomerate; E2-3f = sandstone, calcareous sandstone and limestone; TRn4 = Black limestone, shale and sandstone; I = Massive, recrystalized limestone with minor phyllite and schist) (References: Iran geology organization) (created with ArcGIS version 10.5, https://www.arcgis.com/index.html).

The collected sediment samples from Jazmurian playa were a combination of the 10 × 10 meters samples. Each sample was a combination of 24 surface sediment samples which covered 1600 square meters (Fig. 2). A group of 8 researchers prepared the samples. Each one dug the playa surface with exactly 1 cm thickness at 5 meter intervals using plastic spoons to collect the samples. Then, each person mixed the eight collected sediment samples in a beaker and the sample was dried at 70 °C in an oven. The playa sediments were prepared according to the previous study^6^. In order to obtain a homogenized sample, the sediment sample was ground through a 200-mesh sieve. 12.5 g of the homogenized sample was dissolved in 75 mL aqua regia and then dried. Subsequently, 50 mL solution of nitric acid 2 M was added to the residue and then filtered. The obtained solution was evaporated to 25 mL and consequently diluted to 50 mL. The obtained sample solution was analyzed by ICP- OES. In this study, 24 surface sedimentary samples were collected for heavy metal analysis. The **G**eological map of the study area is illustrated in Fig. 3.

### Elemental analysis

ICP- OES spectrometer (HORIBA Instrument model JY 70 PLUS, Singapore) was applied for elemental analysis of the samples. Prior to the analysis the instrument was calibrated using reference materials QSD (7306, 7309, 7304, 7310, 7311, 7312, 7307, 7305). The instrument was calibrated three times for quality control with standard deviation determined at ±5–7.5%. All stable elements, except for gases, were identified and quantified (Potts, 1987) with total of 23 elements including Fe, Co, Cu, Al, Dy, Pb, Sm, U, Ni, Cd, Cr, Se, Ba, Ti, Mn, V, Sc, Zn, As, Rb, Te, Y and Yb. 0.5 g of each dried sediment was digested in the solution containing nitric acid (65%), hydrochloric acid (37%), perchloric acid (70%), and hydrofluoric acid (48%) using ASTM D4698 method. The solution obtained from sediment preparation was introduced to ICP three times and each concentration value is reported as the mean of three replications.

### Contamination assessment and ecological risks

In order to consider the environmental assessment of Jazmurian playa, a range of pollution indicators, such as geoaccumulation index (Igeo), enrichment factor (EF), pollution load index (PLI) and the potential ecological risk (PER) index were estimated. The mean value of the metal concentrations for each site was applied to estimate each index.

### Degree of contamination index

The term contamination factor C_f_ was applied to describe the contamination of a given toxic substance in a basin:

$${{\rm{C}}}_{{\rm{f}}}={{\rm{C}}}_{{\rm{e}}}/{C}_{b}$$

where Ce is the concentration of the element in sediment samples, and C_b_ is the background values for the element. The degree of contamination, C_d_, is defined as the sum of all contamination factors for various heavy metals. In order to facilitate the pollution control “degree of contamination (Cd)” was proposed by Hakanson which is obtained as follows:

$$Cd=\mathop{\sum }\limits_{i=1}^{i=n}Cf$$

Degree of contamination (mCd) in sediments is defined as:

$$m{C}_{d}=\frac{{\sum }_{i=1}^{i=n}{C}_{f}}{n}$$

In which n is the number of analyzed elements and i=ith element. The clasiifiaction of mCd is shown in Table 1.

**Table 1:** Classification of mCd.

Index	Classification	Sediment contamination	References
mC_d_	mC_d_ < 1.5	very low	^22^
	1.5 < mC_d_ < 2	Low	
	2 < mC_d_ < 4	Moderate	
	4 < mC_d_ < 8	High	
	8 < mC_d_ < 16	Very high	
	16 < mC_d_ < 32	Extremely high	
	32 < mC_d_	Ultra high	

### Geoaccummulation index

Geoaccumulation index (I_geo_) is an indicator which gives assessment of heavy metal contamination of sediments in respect to the background natural levels of the elements. I_geo_ is expressed as follows^22,23^:

$${I}_{geo}={\log }_{2}\frac{{C}_{n}}{1.5\times {B}_{n}}$$

B_n_ and C_n_ are the background and measured concentrations of the sediment samples respectively. Based on the I_geo_ values the samples are classified according to the classification method shown in Table 2.

**Table 2:** Classification of I_geo_ based on the obtained value.

I_geo_	<0	Unpolluted	^30^
	0–1	Unpolluted to moderately polluted	
	1–2	moderately polluted	
	2–3	moderately to strongly	
	3–4	Strongly	
	4–5	strongly to extremely strongly	
	5<	extremely polluted	

### Enrichment factor

Enrichment factor (EF) is another pollution assessment parameter which is widely used to show the enrichment degree and metal contamination of an environmental media. EF normalizes the trace element content with respect to a sample reference metal, such as Fe or Al, as follows^24^:

$$EF=\frac{{(M/Al)}_{SedimentSample}}{{(M/Al)}_{BachgroundSample}}$$

where ${(M/Al)}_{SedimentSample}$ is the ratio of each metal and iron concentration of the sediment sample and ${(M/Al)}_{BackgroundSample}$is ratio of the background sample. The ecological risks based on the EF values are categorized according to Table 3.

**Table 3:** Classification of EF based on the obtained value.

Index	Classification	Sediment contamination	References
EF	≤2	Low enrichment	^31^
	2–5	Moderate enrichment	
	5–20	High enrichment	
	20–40	Very high enrichment	
	>40	Extremely enrichment	

### Pollution load index (PLI)

Pollution load index (PLI) is an index for evaluation of contamination status of sediment samples to heavy metals. PLI is defined as follows^25,26^:

$${\rm{PLI}}={({\rm{CF}}1\times {\rm{CF}}2\times {\rm{CF}}3\times \ldots \mathrm{.}.\times {\rm{CFn}})}^{1/{\rm{n}}}$$

CF is the contamination factor and n is the number of metals.

### Potential ecological risk factor (PER)

Potential ecological risk factor (PER) is a useful index for ecological risk assessments of heavy metals in sediment samples. ER is as follows^27,28^:

$$PE{R}^{i}=\sum {T}_{r}^{i}.{C}_{f}^{i}$$

where $PE{R}^{i}$ is the potential ecological risk factor and ${T}_{r}^{i}$ is the toxic-response factor. The classification of ecological risks according to PER ranges is indicated in Table 4.

**Table 4:** Classification of PER based on the obtained value.

Index	Classification	Sediment contamination	References
PER	PER ≤ 40	low risk	^32^
	40 < PER ≤ 80	moderate risk	
	80 < PER ≤ 160	considerable risk	
	160 < PER ≤ 320	high risk	
	320 < PER	Very high risk	

### GIS analysis

In order to obtain the geochemical mapping, ArcGIS 10.5 was applied. According to the obtained data the spatial distribution of heavy metals was used to predict the un-sampled sites in the study area in which the inverse distance weighting method was used.

### Statistical analysis

SPSS version 25 was also used to analyze Pearson’s correlation coefficient and hierarchical cluster analysis (HCA) to develop groups and identify links among sampling sites. HCA was performed on normalized datasets and presented as dendrogram plotted with linkages between groups using squared Euclidean distance.

## Results and discussion

In this research study 23 elements in 24 sediment samples of Jazmurian Playa were measured. Table 4 displays only the trace metals used to assess the sediment quality based on their threshold guideline values. The elements of concern were compared against the threshold effect level (TEL) below which no adverse effect is observed, and the toxic effect threshold (TET) above which extreme environmental impacts are expected. From all selected sites, site 24 was below TET for all metals, including all TEL values, except for Cr. Because of its lowest concentration of metals, this site was selected as a background site. For all other sites, As and Ni were found to be the metals of the highest importance. All sites exceeded the TEL levels for As, while 15 sites exceeded the TET values demonstrating high level of pollution with As. In case of Ni 15 sites exceeded the TET values, while all other sites, except for 2 sites (2 and 4), exceeded the TEL values. Cr was found above TET levels for site 23 only, with all other sites exceeding the TEL values. In case of Cu, 21 sites exceeded their TEL levels, while 3 sites only (8, 14 and 16) exceeded the Zn TEL levels. All sites were below TEL and TET values for Cd and Pb.

**Figure 4:**
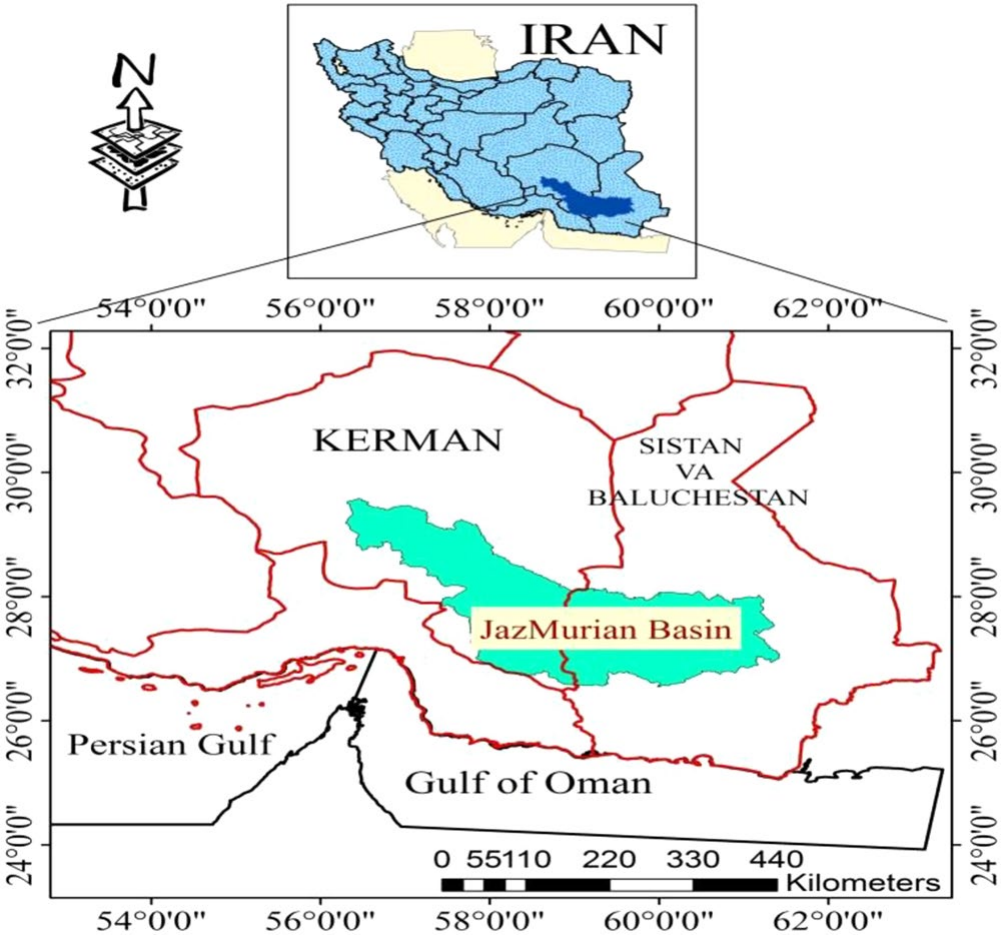
The spatial distribution maps of I_geo_ for (**a**) Pb and (**b**) Ni as strongly polluted elements; (**c**) Cu, and (**d**) Dy as moderately polluted elements.

The selected pollution assessment indices were further investigated. The I_geo_ index for the studied elements is summarized in Tables S1 and S2. Sites 5, 7, 14, 16 and 20 were found moderately to strongly enriched with Pb, while sites 10, 15 and 23 were moderately to strongly polluted with Ni, as determined with the I_geo_ range between 2 and 3. Site 1 was found strongly polluted with Al, with the highest I_geo_ was estimated at 3.24. Other elements had no significant pollution and were categorized as unpolluted (as I_geo_ < 0) or unpolluted to moderately polluted (0 < I_geo_ < 1) elements. A wider range of elements, such as Mn, Y, Zn, Sm, U, Ba, Se, Cu, Co, Dy and Sc also appear in moderate pollution levels at multiple locations throughout the investigated sites. The spacial distribution maps of I_geo_ for Pb and Ni as strongly polluted elements, and Cu and Dy as moderately polluted elements were indicated in Fig. 4.

**Figure 5:**
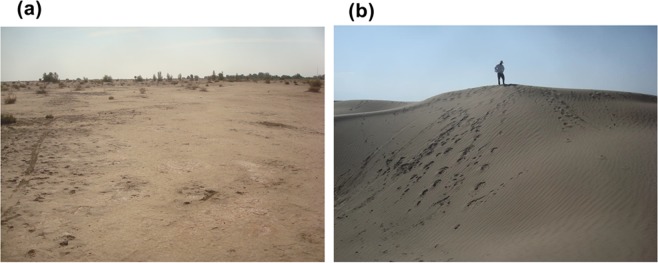
The spatial distribution maps of EF for highly enriched elements of (**a**) Pb, (**b**) Ni, (**c**) Cu, and (**d**) Dy.

The enrichment factor analysis of the elements in the sediment samples of Jazmurian Playa are presented in Table S3. The sediments in sites 5, 7, and sites 12 to 23 were highly enriched with Dy. The sediments of sites 1 to 7, 9, 14 to 16, and 19 to 23 were also highly enriched with Pb. Sites 10, 13, 15, 21 and 23 were highly enriched with Ni and the sediments of site 23 were highly enriched with Cu. Except for As, Cd and Cr all other elements showed moderate enrichment in almost all sites. The classifications of the EF values of each metal in the studied areas are summarized in Table S4. The spatial distribution maps for highly enriched elements of Pb, Ni, Cu, and Dy were indicated in Fig. 5.

In order to show the potential risk of the studied elements, potential ecological risk index (PER) was determined for Cu, Zn, Mn, As, Ni, Cd, Cr and Pb. According to the results presented in Table S4, sites 5, 14, 16 and 20 showed high risk with PER values ranging between 160 and 320. All other sites indicated considerable risk with PER values between 80 and 160.

**Figure 6:**
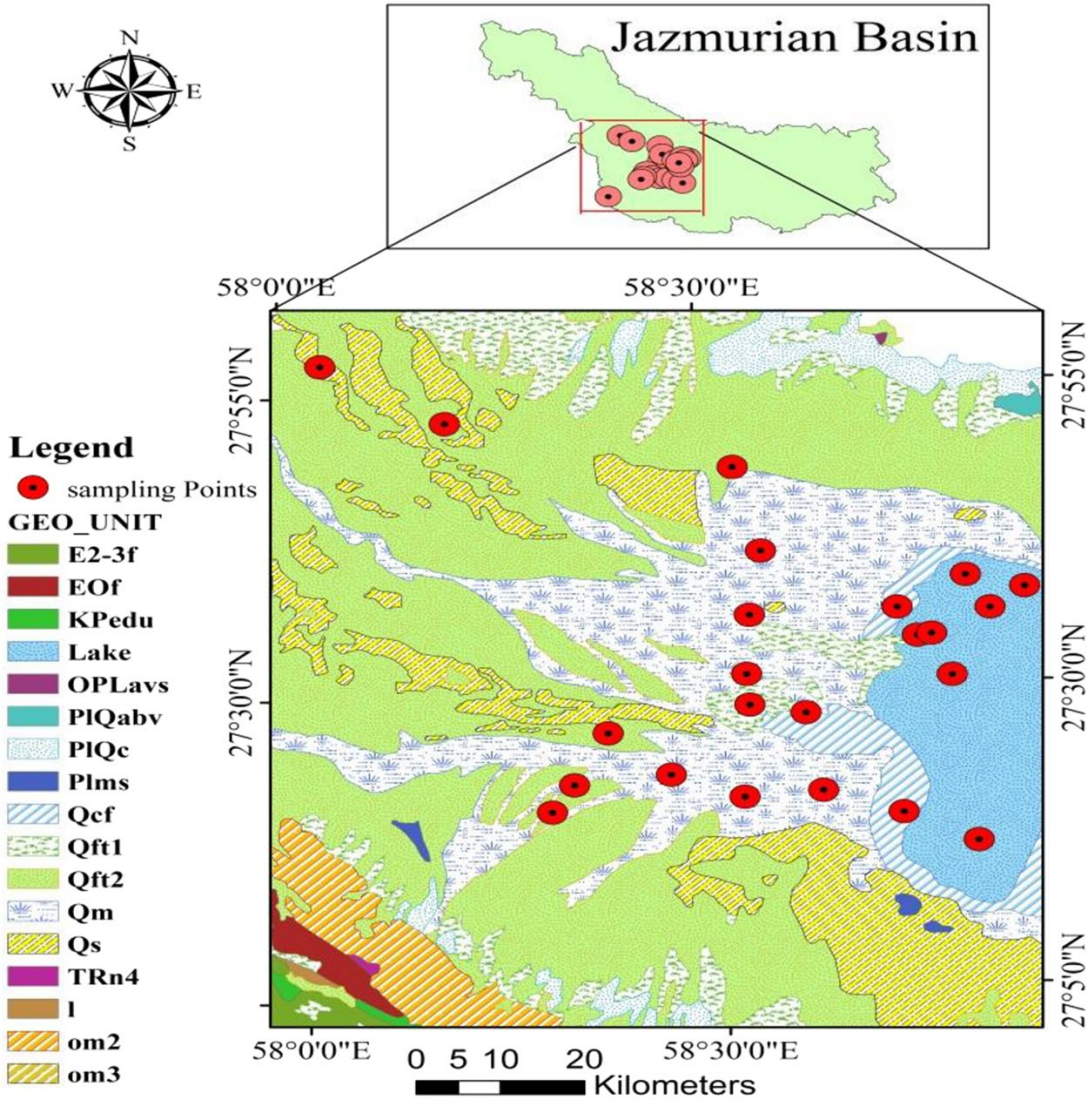
The s spatial distribution maps of (**a**) PER and (**b**) PLI.

The pollution load index (PLI), as an aggregative explanation of the overall level of metal pollution, was investigated and the obtained results shown in Table 5 revealed that all studied areas, except for the background location 24, have PLI values greater than 1, with the highest value of 2.45 for site 15. Figure 6 shows the spacial distribution maps of PER and PLI.

**Table 5:** PER and PLI of different metals in the surface sediments of the study area.

Sites	1	2	3	4	5	6	7	8	9	10	11	12	13	14	15	16	17	18	19	20	21	22	23	24
PER	90.6	100.6	101.9	100.8	162.2	114.3	100.3	117.8	127.9	147.4	113.8	77.6	113.4	165.7	156.8	172.5	74.7	110.2	98.3	182.5	121	121.3	101.9	—
PLI	2.16	1.5	1.89	1.75	2.23	2.09	2.17	2.1	2.03	2.08	1.97	2.1	2.25	2.21	2.45	2.14	1.85	2.2	2.27	2.37	2.33	2.19	2.38	1

Correlation analysis was performed to test the relationship between the elements (Table 6). According to the Pearson statistical analysis Dy shows strong positive correlation with Sm, Te, Co, Se, Fe and Y, while Sm has strong positive correlation with Te, U, Co, Se, Cu, Fe, Mn, Sc, Y and Yb. Te has positive correlation with Co, Cu, Fe, Mn and Y while, U has positive correlation with the same elements except Y. However, Ni is positively correlated with Co, Cu, Fe, Cr, Mn, Sc, Y and Yb, whereas the occurrence of Co is positively correlated with Se, Cu, Fe, Cr, Mn, Sc, Ti, Y and Yb. Cu and Fe are positively correlated with one another as well as with Cr, Mn, Sc, Y and Yb. Besides, Cr is positively correlated with Mn, Sc and Yb. Moreover, Mn and Sc show positive correlation with each other and with Ti, V, Y and Yb. Furthermore, the occurrence of Ti is positively correlated with V, Y and Yb whereas Y and Yb are positively correlated with each other.

**Table 6:** Correlation matrix for trace elements in surface sediments from different sites of the study area.

	*Dy*	*Pb*	*Rb*	*Sm*	*Te*	*U*	*Ni*	*Al*	*Co*	*Ba*	*Se*	*Cu*	*Fe*	*Cd*	*Cr*	*Mn*	*Sc*	*Ti*	*V*	*Y*	*Yb*	*Zn*	*As*
Dy	1.00																						
Pb	0.51	1.00																					
Rb	0.62	0.40	1.00																				
Sm	0.98*	0.49	0.66	1.00																			
Te	0.77*	0.51	0.46	0.82*	1.00																		
U	0.66	0.41	0.37	0.70*	0.62	1.00																	
Ni	0.56	0.13	0.38	0.62	0.55	0.63	1.00																
Al	−0.07	0.04	−0.18	−0.05	−0.16	−0.06	0.06	1.00															
Co	0.82*	0.35	0.51	0.88*	0.79*	0.72*	0.90*	−0.01	1.00														
Ba	0.10	0.19	0.49	0.08	−0.03	0.04	−0.34	−0.04	−0.25	1.00													
Se	0.84*	0.56	0.70	0.89*	0.65	0.67	0.52	0.04	0.74*	0.23	1.00												
Cu	0.77	0.37	0.40	0.81*	0.73*	0.72*	0.86*	0.06	0.92*	−0.26	0.66	1.00											
Fe	0.81*	0.38	0.56	0.87*	0.79*	0.79*	0.78*	0.05	0.91*	−0.02	0.80*	0.87*	1.00										
Cd	−0.01	0.56	0.05	−0.07	0.02	−0.14	−0.23	−0.20	−0.16	0.10	0.01	−0.23	−0.16	1.00									
Cr	0.53	0.11	0.37	0.63	0.54	0.67	0.85*	0.02	0.82*	−0.27	0.60	0.77*	0.73*	−0.38	1.00								
Mn	0.67	0.25	0.25	0.74*	0.72*	0.76*	0.83*	0.06	0.89*	−0.29	0.69	0.88*	0.88*	−0.23	0.83*	1.00							
Sc	0.66	0.22	0.27	0.72*	0.65	0.65	0.93*	0.09	0.94*	−0.46	0.58	0.94*	0.83*	−0.22	0.83*	0.91*	1.00						
Ti	0.60	0.23	0.08	0.63	0.58	0.56	0.57	−0.01	0.72*	−0.52	0.48	0.69	0.72*	−0.18	0.51	0.73*	0.75*	1.00					
V	0.20	−0.09	−0.12	0.30	0.41	0.44	0.67	0.02	0.62	−0.68	0.17	0.58	0.58	−0.31	0.63	0.71*	0.75*	0.80*	1.00				
Y	0.84*	0.43	0.39	0.85*	0.71*	0.65	0.78*	0.08	0.92*	−0.36	0.71	0.89*	0.85*	−0.06	0.67	0.83*	0.91*	0.84*	0.61	1.00			
Yb	0.69	0.27	0.20	0.71*	0.64	0.65	0.79*	0.07	0.85*	−0.51	0.53	0.93*	0.77*	−0.28	0.70*	0.85*	0.92*	0.84*	0.71*	0.91*	1.00		
Zn	0.32	0.50	−0.07	0.33	0.40	0.38	0.44	−0.07	0.50	−0.55	0.23	0.56	0.41	0.19	0.36	0.50	0.62	0.63	0.58	0.62	0.63	1.00	
As	−0.05	−0.12	−0.12	0.03	0.17	0.35	0.19	0.11	0.09	0.11	0.12	0.14	0.26	−0.24	0.34	0.45	0.18	0.00	0.26	0.00	0.09	−0.04	1.00

(Bold)* values are significant at *P* < 0.05.

**Figure 7:**
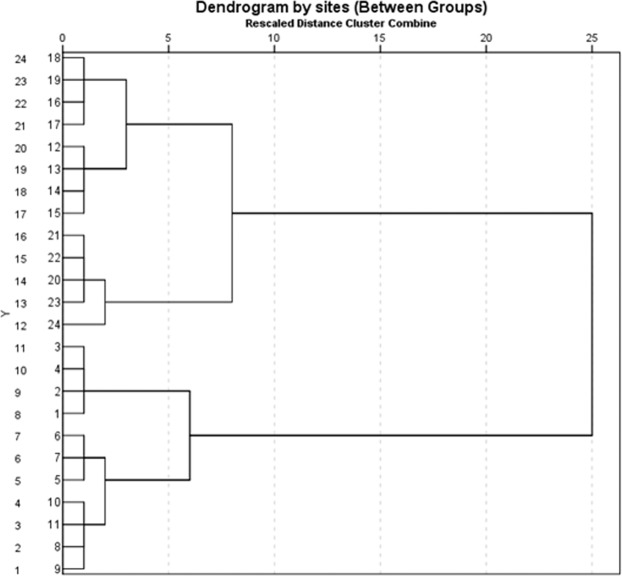
Dendrogram showing cluster of sampling stations based on analyzed variables.

Hierarchical cluster analysis as a multivariate statistical technique was applied on the Squared Euclidean distances and the dendrogram of the sampling sites, as shown in Fig. 7. Dendrogram helps with investigation and understanding of the sampling sites with respect to the analyzed trace element concentrations and their sources^29^. The dendrogram of the sampling sties show two clusters of sampling sites with further sub-clusters. The first cluster comprises three sub-clusters of the sampling sites 12–24 with significant contamination at sites 13, 19 and 20 including the background sites. Cluster 2 includes three sub-clusters of the sampling sites 1–11 with moderate enrichment of trace elements at sites 2, 7 and 11.

All indices (EF, Igeo, PER and PLI) used in the present study specify various levels of contamination in the studied areas although their outcomes are mostly uniform. The reason behind varying level of contamination is that each index assesses different pollutants of importance for their evaluation. For instance, the enrichment factors use terrestrial element (Al in this study) for normalization in the calculation of enrichment factor and appears to provide better contamination assessment than I_geo_, PLI and PER, as it considers more metals and sites. The EF index in this work showed high enrichment of Dy, Pb, Ni and Cu at the study sites 1–7, 9–10, 12–16, 19–23, whereas I_geo_ index identified strong Al pollution followed by moderate to strong pollution of Ni and Pb at sites 1, 5, 7, 10, 14–16, 20. Besides, PER index identified sites 5,14,16, 20 as ecologically high-risk areas which was a result of significant concentrations of Cu, Ni, Cd and Pb. Moreover, PLI values suggested that all the study sites are polluted, with sites 1, 5–10, 12–16 and 18–23 showing substantial pollution levels. The HCA analysis presented as dendrogram also identified 2, 7, 11, 13, 19, 20 as most polluted sites. Except for some variations, all the analysis methods portray overridden results for the study sites that strengthen the rationality of pollution scenarios in the study area. The main attribution to the elevated metals in the studied sites is associated with the existence of different mines which surround the area and transfer the mine soils to Jazmurian bed by seasonal rivers.

## Conclusions

Trace metal contamination in the sediments of 24 sites of Jazmurian playa was analyzed and evaluated by various effective pollution and ecological risk indices. The study results demonstrate that As, Ni and Cr are the metals of great concern in all sites in the study area, significantly at site 15 followed by Cu at site 21 and Zn at sites 8, 14 and 16, as they exceed the threshold guideline values of sediment quality guidelines. The geoaccumulation index (I_geo_) suggests strong Al pollution along with moderate to strong Ni and Pb pollution, whereas the enrichment factor (EF) points on the anthropogenic impacts on the sedimentary environment signifying Dy, Pb, Ni and Cu enrichment in the sediments of the studied areas. Moreover, significant concentrations of Cu, Ni, Cd and Pb in PER index identified four sites (5, 14, 16, 20) as high ecologically-risk areas. Finally, PLI values demonstrate significant pollution at sites 1, 5–10, 12–16 and 18–23 which is further supported by dendrogram. The high concentrations of metal pollution of Jazmurian sediments is associated with the existence of different mines which surround the area. Furthermore, there are seasonal rivers in south Kerman Province which transfer metals from the mines to Jazmurian Playa. This widespread aggregation of metal contaminated soils in Jazmurian has led to the pollution identified in this study. Drying of industrial contaminated river sediments through climate change generates complex phenomena through exposure of the metal enriched sediments to the atmosphere transforming the water to air pollution problem. Climate change will likely lead to related environmental problems in other drought impacted countries, such as Australia, which will need to be taken into consideration when developing national environmental risk assessments of the complex impacts of climate change.

## Supplementary information

Supplementary Information.
