# Supplementary material for: Pollution and contamination assessment of heavy metals in the sediments of Jazmurian playa in southeast Iran
Source: Sci Rep. 2020 Mar 16;10:4775. doi: 10.1038/s41598-020-61838-x (PMC7075869; doi:10.1038/s41598-020-61838-x)
Supplement: Supplementary file 1 — Supplementary Information. [file 41598_2020_61838_MOESM1_ESM.docx]

**Supplementary Information**

**Pollution and contamination assessment of heavy metals in the sediments of Jazmurian playa in southeast Iran**

Mahboube Shirani^a*^, Keramat Nezhad Afzali^b*^, Sayka Jahan^c^, Vladimir Strezov^c,d^, Mojtaba Soleimani-Sardo^e^

^a^ Department of Chemistry, Faculty of Science, University of Jiroft, Jiroft, P. O. Box 7867161167, Iran

^b^ Department of Geography, Faculty of Humanities and Literature, University of Jiroft, Jiroft, P. O. Box 7867161167, Iran

^c^ Department of Earth and Environmental Sciences, Faculty of Science and Engineering, Macquarie University NSW 2109, Australia

^d^ ARC Research Hub for Computational Particle Technology, Macquarie University, Sydney, New South Wales 2109, Australia

^e^ Department of Environmental Science and Engineering, Faculty of Natural Resources, University of Jiroft, Jiroft, P. O. Box 7867161167, Iran

^*^ Corresponding authors: Tel.: +98 3443347061; fax: +98 34 43347065; E-mail addresses: [shirani.mahboubeh@gmail.com](mailto:shirani.mahboubeh@gmail.com), [m.shirani@ujiroft.ac.ir](mailto:m.shirani@ujiroft.ac.ir), and k.nezhadafzali96@gmail.com

| Table S1. I_geo_ of the studied metals of sampling site in the surface sediments of the Jazmurian Playa | | | | | | | | | | | |
| --- | --- | --- | --- | --- | --- | --- | --- | --- | --- | --- | --- |
|  | Elements | | | | | | | | | | |
| Location | **Mn** | **Ti** | **V** | **Y** | **Yb** | **Zn** | **As** | **Rb** | **Sm** | **Te** | **U** |
| 1 | 0.95 | 0.49 | 0.54 | 1.04 | 0.98 | 0.97 | -0.39 | 0.37 | 0.86 | 0.41 | 0.58 |
| 2 | 0.09 | -0.14 | -0.21 | 0.37 | 0.43 | -0.04 | -1.17 | 0.89 | 0.53 | 0 | -0.25 |
| 3 | 0.62 | 0.28 | 0.05 | 0.83 | 0.81 | 0.57 | -0.62 | 0.81 | 0.90 | 0.41 | 0.76 |
| 4 | 0.63 | 0.53 | 0.54 | 0.73 | 0.70 | 0.60 | -0.37 | 0.65 | 0.81 | 0.73 | 0.79 |
| 5 | 1.07 | 0.55 | 0.61 | 1.07 | 1.00 | 1.18 | -0.44 | 0.64 | 1.08 | 1 | 0.94 |
| 6 | 1.01 | 0.48 | 0.52 | 1.001 | 0.98 | 1.08 | -0.38 | 0.52 | 1.00 | 1 | 0.57 |
| 7 | 1.02 | 0.54 | 0.59 | 0.99 | 1.03 | 1.13 | -0.42 | 0.65 | 1.05 | 1 | 0.98 |
| 8 | 1.03 | 0.53 | 0.63 | 1.02 | 0.95 | 1.57 | -0.47 | 0.55 | 1.03 | 0.73 | 0.76 |
| 9 | 0.77 | 0.40 | 0.42 | 0.96 | 0.86 | 1.01 | -0.19 | 0.73 | 0.97 | 0.73 | 0.27 |
| 10 | 1.05 | 0.54 | 0.64 | 1.07 | 1.06 | 1.11 | -0.40 | 0.71 | 0.84 | 0.41 | 0.76 |
| 11 | 0.79 | 0.43 | 0.41 | 0.93 | 0.90 | 0.85 | -0.85 | 0.33 | 0.87 | 0.41 | 1.00 |
| 12 | 0.92 | 0.61 | 0.57 | 1.07 | 1.01 | 1.06 | -1.07 | 0.68 | 1.12 | 0.41 | 0.88 |
| 13 | 1.07 | 0.67 | 0.69 | 1.20 | 1.06 | 1.07 | -1.25 | 0.84 | 1.18 | 1 | 0.48 |
| 14 | 1.006 | 0.69 | 0.58 | 1.17 | 1.08 | 1.62 | -1.23 | 0.62 | 1.08 | 1 | 1.14 |
| 15 | 1.08 | 0.55 | 0.72 | 1.17 | 1.004 | 1.21 | -0.84 | 0.94 | 1.18 | 1 | 0.96 |
| 16 | 0.71 | 0.57 | 0.45 | 1.13 | 1.03 | 1.64 | -1.76 | 0.70 | 1.08 | 0.73 | 0.60 |
| 17 | 0.73 | 0.59 | 0.44 | 1.008 | 0.99 | 0.65 | -1.92 | 0.54 | 1.07 | 0.73 | 0.14 |
| 18 | 1.05 | 0.47 | 0.54 | 1.02 | 1.03 | 1.02 | -0.17 | 0.56 | 1.08 | 1 | 1.03 |
| 19 | 1.05 | 0.54 | 0.64 | 1.08 | 1.05 | 1.11 | -0.20 | 0.73 | 1.12 | 1 | 1.15 |
| 20 | 1.13 | 0.50 | 0.48 | 1.05 | 0.94 | 1.17 | -0.38 | 0.63 | 1.18 | 1 | 1.003 |
| 21 | 1.13 | 0.49 | 0.59 | 1.02 | 1.09 | 1.16 | -0.33 | 0.79 | 1.11 | 1 | 1.02 |
| 22 | 1.22 | 0.59 | 0.61 | 1.06 | 0.99 | 1.08 | 0.21 | 0.50 | 1.10 | 0.73 | 1.01 |
| 23 | 1.17 | 0.64 | 0.76 | 1.14 | 1.11 | 1.16 | -0.38 | 0.92 | 1.20 | 0.73 | 1.28 |
| 24 | 0.37 | 0.33 | 0.64 | 0.64 | 0.71 | 1.10 | 0.10 | 0.12 | 0.63 | 0.41 | -0.58 |

| Table S2. Igeo of the studied metals of sampling site in the surface sediments of the Jazmurian Playa | | | | | | | | | | | | |
| --- | --- | --- | --- | --- | --- | --- | --- | --- | --- | --- | --- | --- |
|  | Elements | | | | | | | | | | | |
| Location | **Ni** | **Cd** | **Al** | **Ba** | **Se** | **Cr** | **Cu** | **Fe** | **Co** | **Dy** | **Pb** | **Sc** |
| 1 | 1.51 | <-1.43 | 3.24 | 0.47 | 0.90 | 0.14 | 1.37 | 0.63 | 0.70 | 1.50 | 1.95 | 1.04 |
| 2 | -0.53 | 0.10 | 0.01 | 1.26 | 0.76 | -0.38 | 0.01 | -0.01 | -0.13 | 1.12 | 1.88 | -0.09 |
| 3 | -0.03 | -0.29 | 0.18 | 1.04 | 0.96 | -0.73 | 0.79 | 0.35 | 0.27 | 1.66 | 1.94 | 0.37 |
| 4 | -0.92 | -0.05 | -0.13 | 1.006 | 0.80 | -0.68 | 0.29 | 0.67 | 0.28 | 1.37 | 1.80 | 0.29 |
| 5 | 1.64 | 0.37 | 0.11 | 0.53 | 0.90 | 0.17 | 1.62 | 0.75 | 0.96 | 1.70 | 2.2 | 1.15 |
| 6 | 1.70 | -1.16 | 0.07 | 0.47 | 0.78 | 0.18 | 1.55 | 0.67 | 0.93 | 1.68 | 1.83 | 1.05 |
| 7 | 1.70 | <-1.43 | 0.14 | 0.86 | 0.87 | 0.12 | 1.61 | 0.70 | 0.92 | 1.78 | 2.09 | 1.03 |
| 8 | 1.55 | -0.66 | 0.13 | 0.64 | 0.95 | 0.26 | 1.52 | 0.74 | 0.84 | 1.68 | 1.66 | 1.14 |
| 9 | 1.08 | 0.15 | 0.06 | 0.69 | 0.75 | -0.03 | 0.96 | 0.48 | 0.59 | 1.69 | 1.77 | 0.81 |
| 10 | 2.11 | 0.10 | 0.15 | 0.40 | 0.88 | 0.16 | 1.63 | 0.76 | 0.83 | 1.55 | 1.56 | 1.20 |
| 11 | 1.45 | -0.16 | 0.12 | 0.58 | 0.61 | 0.16 | 1.15 | 0.48 | 0.65 | 1.68 | 1.38 | 0.89 |
| 12 | 1.50 | <-1.43 | 0.10 | 0.56 | 1.00 | 0.09 | 1.45 | 0.66 | 0.95 | 1.86 | 1.38 | 1.08 |
| 13 | 1.81 | -0.84 | 0.02 | 0.34 | 1.08 | 0.49 | 1.54 | 0.79 | 1.08 | 1.90 | 1.64 | 1.20 |
| 14 | 1.52 | 0.28 | 0.01 | 0.34 | 1.08 | 0.16 | 1.55 | 0.71 | 0.94 | 1.85 | 2.72 | 1.10 |
| 15 | 2.36 | 0.10 | 0.07 | 0.32 | 1.12 | 0.59 | 1.54 | 0.85 | 1.29 | 1.83 | 1.92 | 1.31 |
| 16 | 1.00 | 0.67 | -0.06 | 0.32 | 0.95 | -0.14 | 1.47 | 0.55 | 0.78 | 1.83 | 2.76 | 0.96 |
| 17 | 0.69 | -1.29 | 0.11 | 0.28 | 0.87 | -0.30 | 1.22 | 0.45 | 0.66 | 1.80 | 1.05 | 0.80 |
| 18 | 1.67 | -1.43 | 0.19 | 0.53 | 0.97 | 0.44 | 1.57 | 0.70 | 0.90 | 1.78 | 1.67 | 1.11 |
| 19 | 1.71 | <-1.43 | 0.23 | 0.53 | 0.93 | 0.55 | 1.65 | 0.76 | 0.96 | 1.82 | 1.81 | 1.14 |
| 20 | 1.46 | 0.79 | 0.12 | 0.62 | 1.23 | 0.35 | 1.39 | 0.69 | 0.95 | 1.88 | 2.47 | 1.02 |
| 21 | 1.78 | -1.16 | 0.28 | 0.48 | 1.004 | 0.53 | 1.72 | 0.78 | 1.02 | 1.73 | 1.88 | 1.17 |
| 22 | 1.40 | -0.84 | 0.20 | 0.62 | 1.24 | 0.52 | 1.39 | 0.74 | 0.87 | 1.76 | 1.91 | 1.06 |
| 23 | 2.21 | <-1.43 | 0.29 | 0.47 | 1.18 | 0.96 | 1.77 | 0.88 | 1.16 | 1.79 | 1.77 | 1.27 |
| 24 | -1.21 | -0.75 | 0.19 | 0.46 | 0.00 | 0.00 | 0.00 | 0.00 | 2.06 | -0.58 | 1.26 | 0.33 |

| Table S3. EF of the studied metals of sampling site in the surface sediments of the Jazmurian Playa | | | | | | | | | | | | | | | | | | | | | | |
| --- | --- | --- | --- | --- | --- | --- | --- | --- | --- | --- | --- | --- | --- | --- | --- | --- | --- | --- | --- | --- | --- | --- |
| EF | | | | | | | | | | | | | | | | | | | | | | |
| Location | **Co** | **Dy** | **Pb** | **Rb** | **Sm** | **Te** | **U** | **Mn** | **Sc** | **Ti** | **V** | **Y** | **Ni** | **Cd** | **Ba** | **Se** | **Cr** | **Cu** | **Yb** | **Fe** | **Zn** | **As** |
| 1 | 2.44 | 4.26 | 5.82 | 1.94 | 2.72 | 2 | 2.24 | 2.9 | 3.1 | 2.1 | 2.19 | 3.08 | 4.29 | <0.55 | 2.08 | 2.81 | 1.66 | 3.88 | 2.96 | 2.32 | 2.95 | 1.13 |
| 2 | 1.36 | 3.26 | 5.52 | 2.79 | 2.17 | 1.5 | 1.25 | 1.6 | 1.4 | 1.35 | 1.29 | 1.94 | 1.03 | 1.61 | 3.61 | 2.54 | 1.14 | 1.51 | 2.03 | 1.48 | 1.45 | 0.66 |
| 3 | 1.81 | 4.76 | 5.77 | 2.64 | 2.8 | 2 | 2.55 | 2.3 | 1.94 | 1.82 | 1.55 | 2.67 | 1.46 | 1.22 | 3.08 | 2.91 | 0.9 | 2.6 | 2.63 | 1.91 | 2.24 | 0.97 |
| 4 | 1.82 | 3.9 | 5.24 | 2.36 | 2.63 | 2.5 | 2.59 | 2.33 | 1.84 | 2.17 | 2.19 | 2.5 | 0.78 | 1.44 | 3.01 | 2.62 | 0.93 | 1.84 | 2.44 | 2.39 | 2.28 | 1.15 |
| 5 | 2.92 | 5 | 7.27 | 2.35 | 3.17 | 3 | 2.89 | 3.16 | 3.33 | 2.2 | 2.29 | 3.16 | 4.68 | 1.94 | 2.17 | 2.8 | 1.69 | 4.63 | 3 | 2.53 | 3.4 | 1.09 |
| 6 | 2.86 | 4.81 | 5.35 | 2.15 | 2.98 | 3 | 2.23 | 3.02 | 3.12 | 2.09 | 2.15 | 3.004 | 4.88 | 0.66 | 2.07 | 2.58 | 1.7 | 4.4 | 2.95 | 2.39 | 3.18 | 1.14 |
| 7 | 2.84 | 5.16 | 6.42 | 2.36 | 3.12 | 3 | 2.96 | 3.06 | 3.08 | 2.19 | 2.27 | 2.99 | 4.89 | <0.55 | 2.73 | 2.75 | 1.62 | 4.59 | 3.08 | 2.44 | 3.3 | 1.11 |
| 8 | 2.69 | 4.82 | 4.75 | 2.2 | 3.07 | 2.5 | 2.54 | 3.08 | 3.31 | 2.17 | 2.33 | 3.04 | 4.41 | 0.94 | 2.34 | 2.9 | 1.8 | 4.32 | 2.9 | 2.52 | 4.45 | 1.08 |
| 9 | 2.26 | 4.84 | 5.14 | 2.49 | 2.94 | 2.5 | 1.81 | 2.57 | 2.63 | 1.99 | 2.02 | 2.92 | 3.18 | 1.66 | 2.42 | 2.52 | 1.46 | 2.92 | 2.73 | 2.1 | 3.02 | 1.31 |
| 10 | 2.66 | 4.4 | 4.43 | 2.46 | 2.69 | 2 | 2.54 | 3.12 | 3.44 | 2.18 | 2.34 | 3.16 | 6.51 | 1.61 | 1.99 | 2.77 | 1.67 | 4.65 | 3.14 | 2.54 | 3.25 | 1.13 |
| 11 | 2.37 | 4.81 | 3.9 | 1.88 | 2.75 | 2 | 2.99 | 2.59 | 2.79 | 2.02 | 2 | 2.86 | 4.12 | 1.33 | 2.24 | 2.29 | 1.68 | 3.34 | 2.81 | 2.09 | 2.71 | 0.82 |
| 12 | 2.91 | 5.45 | 3.91 | 2.4 | 3.27 | 2 | 2.76 | 2.85 | 3.17 | 2.29 | 2.23 | 3.16 | 4.25 | <0.55 | 2.22 | 2.99 | 1.59 | 4.09 | 3.03 | 2.36 | 3.13 | 0.7 |
| 13 | 3.18 | 5.62 | 4.68 | 2.69 | 3.42 | 3 | 2.09 | 3.15 | 3.46 | 2.38 | 2.42 | 3.46 | 5.26 | 0.83 | 1.91 | 3.17 | 2.11 | 4.37 | 3.14 | 2.59 | 3.16 | 0.62 |
| 14 | 2.89 | 5.42 | 9.94 | 2.31 | 3.18 | 3 | 3.31 | 3.01 | 3.21 | 2.42 | 2.25 | 3.38 | 4.31 | 1.83 | 1.9 | 3.19 | 1.68 | 4.41 | 3.18 | 2.46 | 4.63 | 0.63 |
| 15 | 3.67 | 5.33 | 5.7 | 2.87 | 3.42 | 3 | 2.93 | 3.19 | 3.73 | 2.2 | 2.48 | 3.39 | 7.72 | 1.61 | 1.87 | 3.26 | 2.26 | 4.37 | 3.01 | 2.71 | 3.47 | 0.83 |
| 16 | 2.59 | 5.36 | 10.16 | 2.45 | 3.19 | 2.5 | 2.28 | 2.45 | 2.92 | 2.23 | 2.05 | 3.28 | 2.99 | 2.38 | 1.87 | 2.9 | 1.35 | 4.15 | 3.07 | 2.2 | 4.7 | 0.44 |
| 17 | 2.37 | 5.23 | 3.12 | 2.19 | 3.15 | 2.5 | 1.65 | 2.5 | 2.62 | 2.26 | 2.04 | 3.01 | 2.42 | 0.61 | 1.83 | 2.75 | 1.21 | 3.49 | 2.97 | 2.06 | 2.35 | 0.39 |
| 18 | 2.81 | 5.17 | 4.77 | 2.21 | 3.18 | 3 | 3.07 | 3.1 | 3.25 | 2.08 | 2.19 | 3.04 | 4.77 | 0.55 | 2.16 | 2.95 | 2.03 | 4.46 | 3.07 | 2.44 | 3.05 | 1.33 |
| 19 | 2.93 | 5.3 | 5.26 | 2.49 | 3.26 | 3 | 3.33 | 3.12 | 3.31 | 2.19 | 2.34 | 3.17 | 4.92 | <0.55 | 2.16 | 2.87 | 2.19 | 4.72 | 3.11 | 2.55 | 3.25 | 1.3 |
| 20 | 2.9 | 5.52 | 8.34 | 2.32 | 3.41 | 3 | 3.007 | 3.29 | 3.04 | 2.12 | 2.09 | 3.11 | 4.13 | 2.61 | 2.3 | 3.52 | 1.91 | 3.95 | 2.88 | 2.42 | 3.38 | 1.15 |
| 21 | 3.04 | 5 | 5.51 | 2.6 | 3.25 | 3 | 3.04 | 3.28 | 3.39 | 2.11 | 2.27 | 3.05 | 5.15 | 0.66 | 2.1 | 3.01 | 2.17 | 4.97 | 3.2 | 2.59 | 3.35 | 1.18 |
| 22 | 2.74 | 5.1 | 5.6 | 2.12 | 3.22 | 2.5 | 3.02 | 3.5 | 3.14 | 2.26 | 2.29 | 3.13 | 3.98 | 0.83 | 2.31 | 3.56 | 2.15 | 3.94 | 2.98 | 2.51 | 3.17 | 1.74 |
| 23 | 3.35 | 5.18 | 5.14 | 2.85 | 3.45 | 2.5 | 3.64 | 3.38 | 3.64 | 2.35 | 2.54 | 3.31 | 6.96 | <0.55 | 2.08 | 3.4 | 2.93 | 5.11 | 3.24 | 2.77 | 3.36 | 1.15 |
| 24 | 1 | 1 | 3.6 | 1.63 | 2.32 | 2 | 1 | 1.93 | 1.88 | 1.88 | 2.34 | 2.04 | 0.68 | 0.88 | 20.6 | 1.97 | 1.55 | 2.04 | 2.46 | 1.82 | 3.23 | 1.61 |

| Table S4. Total classification of EF values of each metal in studied areas | | | | | | | | | | | | | | | | | | | | | | | | |
| --- | --- | --- | --- | --- | --- | --- | --- | --- | --- | --- | --- | --- | --- | --- | --- | --- | --- | --- | --- | --- | --- | --- | --- | --- |
| Elements | **Locations** | | | | | | | | | | | | | | | | | | | | | | | |
|  | **1** | **2** | **3** | **4** | **5** | **6** | **7** | **8** | **9** | **10** | **11** | **12** | **13** | **14** | **15** | **16** | **17** | **18** | **19** | **20** | **21** | **22** | **23** | **24** |
| Au | L^a^ | L | L | L | L | L | L | L | L | L | L | L | L | L | M^b^ | L | L | L | L | L | L | L | L | L |
| Co | M | L | L | L | M | M | M | M | M | M | M | M | M | M | M | M | M | M | M | M | M | M | M | L |
| Dy | M | M | M | M | H^c^ | M | H | M | M | M | M | H | H | H | H | H | H | H | H | H | H | H | H | M |
| Pb | H | H | H | H | H | H | H | M | H | M | M | M | M | H | H | H | M | M | H | H | H | H | H | M |
| Rb | L | M | M | M | M | M | M | M | M | M | L | M | M | M | M | M | M | M | M | M | M | M | M | L |
| Sm | M | M | M | M | M | M | M | M | M | M | M | M | M | M | M | M | M | M | M | M | M | M | M | M |
| Te | L | L | L | M | M | M | M | M | M | L | L | L | M | M | M | M | M | M | M | M | M | M | L | L |
| U | M | L | M | M | M | M | M | L | M | M | M | M | M | M | M | M | L | M | M | M | M | M | M | M |
| Ni | M | L | L | L | M | M | M | M | M | H | M | M | H | M | M | M | M | M | M | M | H | M | H | L |
| Cd | L | L | L | L | L | L | L | L | L | L | L | L | L | L | L | M | L | L | L | M | L | L | L | L |
| Ba | M | M | M | M | M | M | M | M | M | L | M | M | L | L | L | L | L | M | M | M | M | M | M | M |
| Se | M | M | M | M | M | M | M | M | M | M | M | M | M | M | M | M | M | M | M | M | M | M | M | L |
| Cr | L | L | L | L | L | L | L | L | L | L | L | L | M | L | M | L | L | M | M | L | M | M | M | L |
| Cu | M | L | M | L | M | M | M | M | M | M | M | M | M | M | M | M | M | M | M | M | M | M | H | M |
| Fe | M | L | L | M | M | M | M | M | M | M | M | M | M | M | M | M | M | M | M | M | M | M | M | L |
| Mn | M | L | M | M | M | M | M | M | M | M | M | M | M | M | M | M | M | M | M | M | M | M | M | L |
| Sc | M | M | M | M | M | M | M | M | M | M | M | M | M | M | M | M | M | M | M | M | M | M | M | L |
| Ti | M | L | L | M | M | M | M | M | M | M | M | M | M | M | M | M | M | M | M | M | M | M | M | L |
| V | M | L | L | M | M | M | M | M | M | M | M | M | M | M | M | M | M | M | M | M | M | M | M | M |
| Y | M | L | M | M | M | M | M | M | M | M | M | M | M | M | M | M | M | M | M | M | M | M | M | M |
| Yb | M | M | M | M | M | M | M | M | M | M | M | M | M | M | M | M | M | M | M | M | M | M | M | M |
| Zn | M | L | M | M | M | M | M | M | M | M | M | M | M | M | M | M | M | M | M | M | M | M | M | M |
| As | L | L | L | L | L | L | L | L | L | L | L | L | L | L | L | L | L | L | L | L | L | L | L | L |
| ^a^ L for Low enrichment; b M for Moderate enrichment; c H for High enrichment | | | | | | | | | | | | | | | | | | | | | | | | |
